# Novel Bioactive Glass-Modified Hybrid Composite Resin: Mechanical Properties, Biocompatibility, and Antibacterial and Remineralizing Activity

**DOI:** 10.3389/fbioe.2021.661734

**Published:** 2021-06-01

**Authors:** Xiao Han, Yan Chen, Qian Jiang, Xin Liu, Yaming Chen

**Affiliations:** ^1^Jiangsu Key Laboratory of Oral Diseases, Nanjing Medical University, Nanjing, China; ^2^Department of Polyclinics, Affiliated Hospital of Stomatology, Nanjing Medical University, Nanjing, China; ^3^Department of Periodontology, Shanghai Key Laboratory of Craniomaxillofacial Development and Diseases, Shanghai Stomatological Hospital, Fudan University, Shanghai, China; ^4^Department of Oral Surgery, Shanghai Ninth People’s Hospital, Shanghai Jiao Tong University School of Medicine, College of Stomatology, Shanghai Jiao Tong University, National Center for Stomatology, National Clinical Research Center for Oral Diseases, Shanghai Key Laboratory of Stomatology, Shanghai, China; ^5^Department of Dental Materials, Shanghai Biomaterials Research & Testing Center, Shanghai Ninth People’s Hospital, Shanghai Jiao Tong University School of Medicine, College of Stomatology, Shanghai Jiao Tong University, National Center for Stomatology, National Clinical Research Center for Oral Diseases, Shanghai Key Laboratory of Stomatology, Shanghai, China

**Keywords:** composite resin, bioactive glass, antibacterial activity, remineralization, biocompatibility

## Abstract

Secondary caries seriously limits the lifetime of composite resin. However, integrating all desirable properties (i.e., mechanical, antibacterial, bioactivity, and biocompatibility) into one composite resin is still challenging. Herein, a novel bioactive glass (BAG)-modified hybrid composite resin has been successfully developed to simultaneously achieve excellent mechanical properties, good biocompatibility, and antibacterial and remineralizing capabilities. When the mass fractions of BAG particles were added from 8 to 23 wt %, the original mechanical properties of the composite resin, including flexural strength and compressive strength, were not obviously affected without compromising the degree of conversion. Although the BAG incorporation of mass fractions of 16 wt % to 23 wt % in composite resins reduced cell viability, the viability could be recovered to normal by adjusting the pH value. Moreover, the BAG-modified composite resins that were obtained showed good antibacterial effects against *Streptococcus mutans* and enhanced remineralizing activity on demineralized dentin surfaces with increasing incorporation of BAG particles. The possible mechanisms for antibacterial and remineralizing activity might be closely related to the release of bioactive ions (Ca^2+^, Si^4+^), suggesting that its antibacterial and biological properties can be controlled by modulating the amounts of bioactive ions. The capability to balance the mechanical properties, cytotoxicity, antibacterial activity, and bioactivity makes the BAG-modified composite resin a promising prospect for clinical application. Our findings provide insight into better design and intelligent fabrication of bioactive composite resins.

## Introduction

Dental caries is one of the most prevalent bacterial infectious diseases worldwide and usually leads to defects in dental hard tissue. Over the past decades, due to its unique advantages such as simple operation, minimal invasiveness, and excellent esthetic restorative effects, direct composite resin filling has been the main treatment method to repair dental hard tissue defects. However, a higher annual failure rate has been reported for posterior composite resin restorations than for amalgam restorations, which is attributed primarily to secondary caries (Kopperud et al., 2012; Opdam et al., 2014; Astvaldsdottir et al., 2015). Generally, secondary caries occur on the tooth after composite resin filling because oral biofilm bacteria are more likely to accumulate on the surface of resin restorations, leading to demineralization of dental hard tissues due to acid production, thus destroying the tooth structure and further providing a way for bacterial invasion (Arun et al., 2021). Since secondary caries limit the lifetime of composite resin, it is urgent to endow composite resin with antibacterial properties and remineralizing capability. However, currently, integrating multiple desirable properties (e.g., high strength, antibacterial activity, remineralizing ability, and biocompatibility) into one composite resin is still challenging.

Over the past decade, to combat secondary caries, numerous efforts have been devoted to synthesizing various antibacterial monomers with quaternary ammonium salts or incorporating antibacterial drugs/fillers [e.g., chlorhexidine, fluoride, zinc oxide (ZnO), and silver (Ag)] into the resin matrix to improve the antibacterial properties (Cheng et al., 2012; Zhang et al., 2014; Cocco et al., 2015; Farrugia and Camilleri, 2015; Arun et al., 2021). However, the antibacterial effect of Ag- or ZnO nanoparticle (NP)-based composite resins has been reported not to be long-lasting, and the release of antibacterial agents (e.g., monomer/drug/Ag^+^) would impair the mechanical properties and increase the risk of damage to human health. Moreover, these antibacterial monomer/drug/metal oxide NPs have no remineralizing ability, largely impeding their development as composite resins (Cocco et al., 2015; Arun et al., 2021). Recently, promising alternatives such as bioactive ceramic particles [e.g., calcium phosphate nanoparticles (NACPs), bioactive glass (BAG), zinc-doped phosphate-based glass] have been exploited as inorganic fillers to construct orthodontic adhesives or composite resins, which have been found to achieve both antibacterial and remineralizing effects (Liu et al., 2018; Par et al., 2019b; Bhadila et al., 2020; Lee et al., 2020). Among bioactive ceramic particles, BAG has attracted increasing interest in dental applications such as toothpaste, dentin desensitizers, orthodontic adhesives, and experimental composite resins (Wang et al., 2010; Yang et al., 2016; Al-Eesa et al., 2019; Aponso et al., 2019; Par et al., 2019a). In particular, BAG-based composites have been reported to inhibit bacterial biofilm penetration into marginal gaps of simulated tooth restorations, showing great potential to eliminate secondary tooth decay after composite resin restorations (Khvostenko et al., 2016). Nevertheless, the incorporation of bioactive materials may reduce the physical properties of composite resin and cause toxic effects (Kurata et al., 2011; Korkut et al., 2016; Cheng et al., 2017). Despite a few studies focused on developing BAG-based bioactive composite resins (Korkut et al., 2016), it is still unclear whether the antibacterial effect is dependent on direct contact with bacteria or the release of substances (e.g., Ca^2+^ or Si^4+^ ions). In addition, the potential risk for the application of BAG to composite resin remains elusive. To date, a systematic understanding of the influence of precise composition control of BAG fillers on their mechanical, antibacterial, remineralizing, and biological effects is still lacking. More importantly, the related antibacterial, remineralizing, and biological mechanisms of BAG-modified composite resins have not been completely elucidated, making it difficult to further improve their mechanical performance and enhance their antibacterial and remineralizing activities.

Herein, in this work, we developed a novel bioactive hybrid composite resin with good mechanical properties, excellent biocompatibility, and antibacterial and remineralizing activity. Subsequently, the regulatory effects of BAG incorporation on the comprehensive properties of the as-prepared composite resins were systematically investigated. Moreover, a possible antibacterial, remineralizing, and cytotoxicity mechanism of bioactive composite resins was proposed based on ion release and pH value. Overall, the BAG-modified composite resins obtained were highly promising composite resin systems for achieving both antibacterial and remineralizing activity with low cytotoxicity, providing insights into better design and intelligent fabrication of bioactive composite resins.

## Materials and Methods

### Preparation of BAG-Modified Hybrid Composite Resin

The experimental BAG-modified composite resins were prepared by mixing the resin matrix and inorganic fillers containing various mass fractions of BAG particles (0, 8, 16, and 23%) by DMG Dental Material Gesellschaft mbH (Hamburg, Germany). As shown in Table 1, the as-prepared composite resins had filler/resin ratios similar to the ratios used in commercial composite resin systems (EcuSphere^TM^; DMG, Hamburg, Germany). The matrix resins were formulated from commercially available monomers and the components of the photoinitiator system (EcuSphere^TM^; DMG, Hamburg, Germany). The surface silanized barium glass filler and the fumed silica were formulated from commercially available inorganic fillers (EcuSphere^TM^; DMG), and 45S5 BAG particles with an average particle size of 7.26 μm were provided by Beijing Datsing Bio-Tech Co., Ltd. (Beijing Datsing Company, Beijing, China). Briefly, the photoinitiator and organic amine activator were added to the resin monomer mixture and then the viscous resin matrix was obtained. Subsequently, BAG particles were incorporated into inorganic fillers at 0, 8, 16, and 23 wt % by replacing the same amount of silanized barium glass filler with a total filler load maintained at 76 wt %, and then the inorganic fillers were mixed with the resin matrix using a centrifugal mixing device (Speed-Mixer DAC 150 FVZ, Hauschild, Germany). Finally, four kinds of BAG-modified composite resins were prepared, which were designated BAG0 (control), BAG8, BAG16, and BAG23 according to the weight fractions of incorporated BAG particles. Microstructural characterization and element analysis of the as-prepared composite resins were examined using scanning electron microscopy (SEM) with energy-dispersive X-ray spectroscopy (EDS) mapping (Mira3, Tescan, Czechia).

**TABLE 1:** The composition of different parameters for BAG-modified composite resin formulations.

**Materials**	**Resin^*a*^ (wt.%)**	**Total filler (wt.%)**		
		**Barium glass^*b*^**	**Bioactive glass**	**Fumed silica**
BAG0	24%	73%	0%	3%
BAG8	24%	65%	8%	3%
BAG16	24%	57%	16%	3%
BAG23	24%	50%	23%	3%

*^*a*^EcuspHere resin^*TM*^ containing photoinitiator and stabilizer provided by the manufacturer.*

*^*b*^The average particle size of barium glass fillers is ∼0.7 μm.*

### Flexural Strength Testing

According to the requirement of ISO 4049 (Standardization IOF, 2019), bar-shaped test specimens (25 × 2 × 2 mm) were fabricated in stainless-steel split molds and cured by visible light in five overlapping sections on each upper and lower side. After 24-h storage in distilled water at 37°C, five specimens for each composite type were conducted to a three-point bending test using a universal testing machine (Lloyd Instruments Ltd., Fareham Hants, United Kingdom) at a cross-head speed of 0.5 mm/min. Flexural strength was calculated in megapascals (MPa) with the following equation:

(1) $$\sigma_{\text{f}}=\frac{3\,P\,L}{2\,b\,d\,2}$$

where σ_*f*_ is the flexural strength, *P* is the load at fracture (N), *L* is the specimen span (mm), *b* is the specimen width (mm), and *d* is the specimen height (mm).

### Compressive Strength Testing

Cylindrical-shaped test specimens with 4 mm in diameter and 6 mm in height were prepared in stainless-steel split molds and cured by visible light in five overlapping sections on each upper and lower side and stored in water at 37°C prior to test. After 24 h, five specimens from each composite type were tested using a universal testing machine (Lloyd Instruments Ltd., Fareham Hants, United Kingdom) at a cross-head speed of 1 mm/min. Compressive strength was determined in MPa with the following equation:

(2) $$\sigma_{\text{c}}=\frac{P}{S}=\frac{P}{\pi \times r^{2}}$$

where σ_*c*_ is the compressive strength, *P* is the maximum load at fracture (N), *S* is the specimen cross-section area (mm^2^), and *r* is the specimen cross-sectional radius (mm).

### Microhardness Testing

Five disk-shaped specimens 6 mm in diameter and 4 mm in thickness were prepared for each composite resin group. The Vickers microhardness of each composite type was measured on the test specimen surface by a microhardness tester (Shanghai Taiming Optical Instrument Co., Ltd., Shanghai, China) with a 50 gf load (0.490 N) for 15 s. The Vickers microhardness was calculated from the expression HV = 0.1891 *F*/*d*^2^, where HV is the Vickers hardness, *F* is the test load (N), and *d* is the mean value of the indentation’s diagonal lengths (μm).

### Degree of Conversion

The as-prepared composite samples (*n* = 3/group) were completely cured by visible light for 30 s. The uncured samples were pressed into KBr pellets (*d* = 1 cm) using spectroscopically pure KBr. The DC was determined by a Fourier transform infrared spectrometer (IR/Nicolet 6700, Thermo Fisher, United States). The spectra of unpolymerized and polymerized composite specimens were recorded at room temperature, corrected by subtracting the background and then converted into the absorbance mode. The DC (%) was calculated using the relative change in the peak height of the spectral band at 1638 cm^–1^ (aliphatic C = C stretching), and the band at 1610 cm^–1^ (aromatic C = C stretching) was used as a reference. DC was calculated according to the following equation:

$$DC\left(\%\right)=\left(1-\frac{{\left(\frac{1638\,c\,m^{-1}}{r\,e\,f\,e\,r\,e\,n\,c\,e}\right)}_{p\,e\,a\,k\,h\,e\,i\,g\,h\,t\,a\,f\,t\,e\,r\,c\,u\,r\,i\,n\,g}}{{\left(\frac{1638\,c\,m^{-1}}{r\,e\,f\,e\,r\,e\,n\,c\,e}\right)}_{p\,e\,a\,k\,h\,e\,i\,g\,h\,t\,b\,e\,f\,o\,r\,e\,c\,u\,r\,i\,n\,g}}\right)\times 100\%$$

### Agar Diffusion Test for Cytotoxicity

According to ISO 10993-5 and ISO 7045 (Standardization IOF, 2009, 2011), the agar diffusion test as a cytotoxicity barrier testing method was performed for the non-specific cytotoxicity of the leachable components of the as-prepared composite specimens after diffusion through agar. Disk-shaped specimens 6 mm in diameter and 4 mm in thickness were used for cytotoxicity analysis. In detail, L929 cells (Cell Bank of the Chinese Academy of Sciences, Shanghai, China) were propagated in Eagle’s minimum essential medium (MEM) (Gibco, United States) with 10% fetal bovine serum (FBS) (Gibco, United States). Cell suspensions (2.5 × 10^5^ cells/ml) were seeded in cell culture dishes (Corning, NY, United States) and incubated at 37°C with 5% CO_2_. After 24 h, the medium was replaced with 10 ml of freshly prepared agar medium containing 2 × MEM, and then 10 ml of neutral red solution (0.01% in phosphate-buffered saline, San Aisi Co., Ltd., Shanghai, China) was added to the solidified culture medium in the dark for 20 min. The test specimens were placed on the agar surface along with the positive (polyvinylchloride containing organotin additive sheet) and negative controls (high-density polytetrafluoroethylene sheet) in the same cell culture dish. After 24 h of incubation, as shown in Tables 2–4, the decolorization index, lysis index, and cytotoxicity scoring grade were assessed under an optical microscope (Olympus, Japan) according to ISO 7405.

**TABLE 2:** The decolorization index.

**The decolorization index**	**Description**
0	No decolorization
1	Decolorization only under the specimen
2	Decolorization in a zone not greater than 5 mm from the specimen
3	Decolorization in a zone not greater than 10 mm from the specimen
4	Decolorization in a zone greater than 10 mm from the specimen
5	The total culture is decolorized

**TABLE 3:** The cell lysis index.

**The cell lysis index**	**Description**
0	No cell lysis detectable
1	Less than 20% cell lysis
2	20–40% cell lysis
3	40–60% cell lysis
4	60–80% cell lysis
5	Greater than 80% cell lysis

**TABLE 4:** Cytotoxicity scoring grade.

**The scoring grade**	**Cell response^*a*^**	**Description**
0	0	Non-cytotoxic
1	1	Slightly cytotoxic
2	2–3	Moderately cytotoxic
3	4–5	Severely cytotoxic

*^*a*^Cell response scored by the median of the decolorization index and the cell lysis index from each group conducted in quadruplicate.*

### MTT Assay for Cytotoxicity

According to ISO 10993.5 and ISO 10993.12 (Standardization IOF, 2009, 2012), disk-shaped specimens 6 mm in diameter and 4 mm in thickness were fabricated. To prepare the extracts of the experimental composite resins, the as-prepared composite specimens were fully immersed in MEM with 10% FBS under sterile conditions at 37°C for 24 h based on a ratio of 3 cm^2^/ml [surface area of the sample to volume of extraction vehicle]. The extracts were diluted by twofold serial dilutions in the culture medium and resulted in the following concentrations: 12.5, 25, 50, and 100%. The pH value of the extract of each type of composite resin was measured with an ultrabasic benchtop pH meter (Denver Instrument Co., United States). To clarify the role of pH values on the cytotoxicity, 1 N HCl was added into the extracts slowly, with stirring, to adjust the pH value of the extracts to neutral. Cytotoxicity was assessed by using the MTT assay. Briefly, L929 cells were seeded in 96-well plates for 24 h and then treated with different concentrations of extracts of four kinds of experimental composite resins at 12.5, 25, 50, and 100% for 24 h. Following 24 h of treatment, MTT solution (20 μl, 5 mg/ml) (Amresco, Solon, OH, United States) was added to each well and incubated for an additional 4 h in a 37°C incubator. Subsequently, 150 μl of DMSO was added to dissolve the formazan crystals. The absorbance at 570 nm and 630 nm was measured by a microplate reader (Multiskan GO, Thermo Scientific, Waltham, MA, United States).

### Determination of Ion Release and Antibacterial Activity

The antibacterial activity of four experimental composite resins was evaluated using both direct contact and indirect methods of measuring the antibacterial effects of extracts. *Streptococcus mutans* (UA159) was obtained from Shanghai Key Laboratory of Stomatology, Ninth People’s Hospital, Shanghai Jiao Tong University School of Medicine (Shanghai, China). Disk-shaped specimens 6 mm in diameter and 4 mm in thickness were used for antibacterial activity measurement. In detail, *S. mutans* (UA159) at a density of 1 × 10^6^ colony-forming units (CFU)/ml in 0.2 ml was seeded on the surface of as-prepared composite specimens in a sterilized 24-well plate containing brain heart infusion (BHI) (1.8 ml/well) (Difco Laboratories, United States) at 37°C under standard anaerobic conditions (80% N_2_, 10% H_2_, 10% CO_2_) for 24 h. After incubation for 24 h, the bacterial suspension was collected from the experimental composite resins. Following dilution with BHI, 0.1 ml of bacterial suspension was placed on a BHI agar plate and further incubated for 24 h; subsequently, the number of bacterial CFU was counted according to previous studies (He et al., 2015; Yu et al., 2017). Furthermore, to determine whether the factors released by the experimental composite resin had antibacterial effects, each type of composite resin specimen was immersed in BHI (2 ml) for 24 h and 72 h. Subsequently, the BHI extracts of composite resins were collected to culture *S. mutans* for 24 h. Then, the bacterial suspension was diluted and placed on agar plates. The bacterial CFU were counted after culturing for 24 h. *S. mutans* was seeded on each type of composite resin at 37°C under standard anaerobic conditions. After being cultured for 24 h, the bacterial samples on the composite resin were fixed with 2% glutaraldehyde, followed by progressive dehydration with anhydrous ethanol, critical-point drying, and gold spraying. The bacterial morphology and adhesion were characterized by SEM (Hitachi S-3400N, Hitachi, Japan). In addition, the concentrations of Si and Ca in the BHI extracts of the as-prepared composite resin specimens after 24 and 72 h were measured by inductively coupled plasma mass spectrometry (ICP-MS) (Agilent Technologies 5100, Agilent Technologies Co., Ltd., United States).

### Determination of Dentin Remineralization

Dentin remineralization was studied *in vitro* using simulated body fluid (SBF). SBF with a pH of 7.4 was prepared by dissolving the reagent chemicals NaCl, NaHCO_3_, KCl, K_2_HPO_4_⋅3H_2_O, MgCl_2_⋅6H_2_O, CaCl_2_, and Na_2_SO_4_ in deionized water according to a previous study (Mariano et al., 2009). Disk-shaped resin specimens (diameter: 6 mm, height: 4 mm) with different loadings of BAG were prepared. Human teeth were extracted, and the first premolars for orthodontic treatment needs from patients at the Department of Oral Surgery, Ninth People’s Hospital, Shanghai Jiao Tong University School of Medicine were collected and cleaned. The study was approved by the Ethics Committee of Ninth People’s Hospital, Shanghai Jiao Tong University School of Medicine. Each tooth was sectioned to obtain 1-mm-thick dentin disks along its horizontal axis below the enamel–dentin junction using a hard tissue slicer (Leica SP1600, Leica, Germany). Prior to use in the experiment, all dentin disk specimens were washed with 0.5% NaClO for 5 min and sterilized with an excess of deionized water and 70% ethanol for 20 min. Subsequently, all dentin disk specimens were completely demineralized in 10% phosphoric acid for 12 h at 25°C (Tezvergil-Mutluay et al., 2017). Each type of cured composite resin specimen was immersed with one demineralized dentin disk specimen in SBF at pH 7.4 and 37°C for 21 days without changing the aqueous medium (Xie et al., 2008). After immersion, all dentin samples were dried at room temperature and cleaned with acetone, and then the dentine surface morphology was observed and analyzed by SEM-EDS (Mira3, Tescan, Czechia).

## Results

### Characterization of Physicochemical and Mechanical Properties of BAG-Modified Composite Resin

**FIGURE 1:**
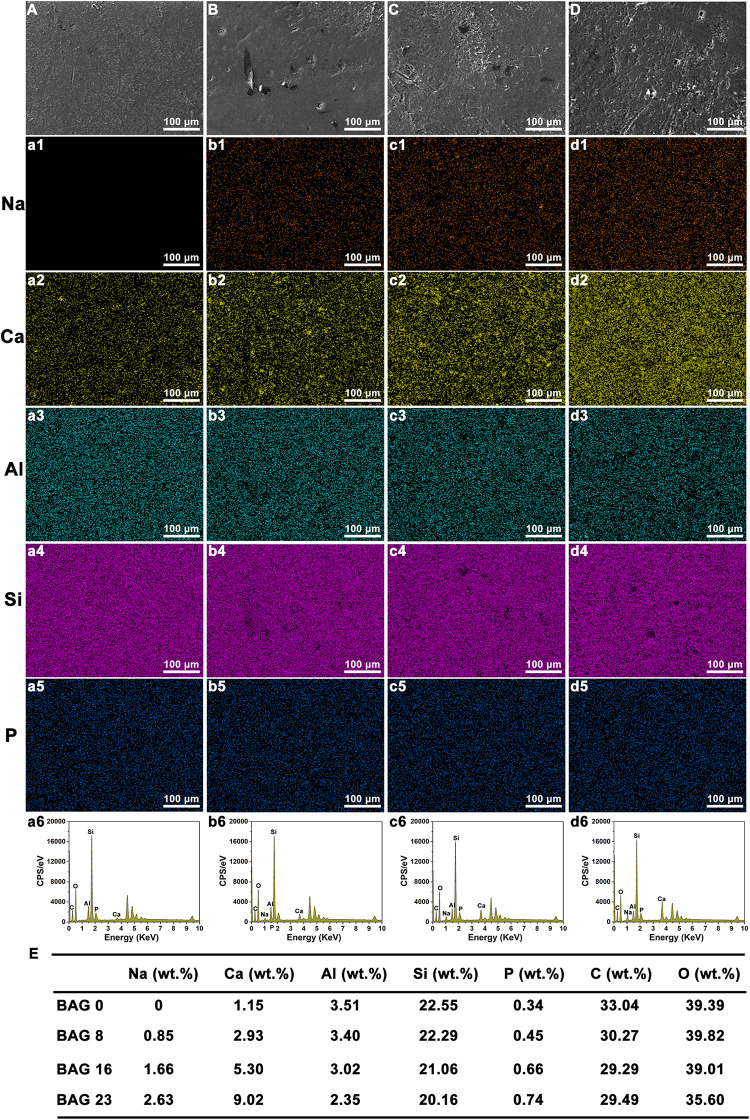
Scanning electron microscopy (SEM) with energy-dispersive X-ray spectroscopy (EDS) mapping analysis of bioactive glass (BAG)-modified composite resins. **(A–D)** SEM images; EDS mapping analysis of Na element **(a1–d1)**; Ca element **(a2–d2)**; Al element **(a3–d3)**; Si element **(a4–d4)**; P element **(a5–d5)**. **(A, a1–a6)** BAG0 group; **(B, b1–b6)** BAG8 group; **(C, c1–c6)** BAG16 group; **(D, d1–d6)** BAG23 group; **(E)** Quantitative analysis of various elements in BAG-modified composite resins. BAG0 indicates composite resin without BAG; BAG8 indicates composite resin with 8 wt% BAG; BAG16 indicates composite resin with 16 wt% BAG; BAG23 indicates composite resin with 23 wt% BAG.

**FIGURE 2:**
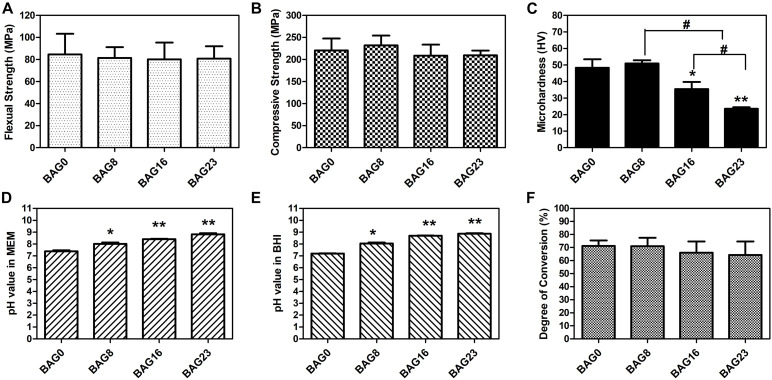
Physicochemical and mechanical properties of bioactive glass (BAG)-modified composite resins. **(A)** Flexural strength. **(B)** Compressive strength. **(C)** Microhardness. The changes in pH values in the extracts of various BAG-modified composite for 24 h in MEM **(D)** and in BHI **(E)**. **(F)** Degree of conversion. MEM: the extracts of various BAG-modified composite resin in MEM with 10% FBS; BHI: the extracts of various BAG-modified composite resin in brain heart infusion (BHI). All the data represent the mean ± SD (*n* = 5); *p < 0.05, **p < 0.01 vs. the BAG0 group; #*p* < 0.05 significant difference as compared groups.

As shown in Figure 1, SEM-EDS elemental mapping showed a uniform elemental distribution of Ca, Na, and Si in the BAG-modified composite resins, and the relative content of each element in the experimental composite resins increased with increasing BAG content. After immersion in deionized water for 24 h, the flexural strength and the compression strength in the composite resin were not obviously reduced by the addition of up to 23 wt % BAG particles (Figures 2A,B). When the mass ratio of BAG particles incorporated into the composite was 8 wt %, there was no significant difference in the microhardness between the BAG8 group (50.9 ± 1.93) and the control group (48.33 ± 5.18) (*p* > 0.05). When the mass ratio of incorporated BAG particles was increased to 16 and 23 wt %, the microhardness was significantly lower than the microhardness of the control group and the BAG8 group (*p* < 0.05) (Figure 2C). Furthermore, after immersion in cell or bacterial culture medium for 24 h, both extracts of composite resin without BAG particles exhibited a pH between 7.2 and 7.4 and increased to 8.8–8.9 for the BAG23 groups, showing a dose-dependent increase in the pH value with increasing BAG incorporation (Figures 2D,E). Moreover, the degree of conversion (DC) value was not affected in the experimental resin system by increasing the BAG amounts (Figure 2F).

### Biocompatibility Assessment of BAG-Modified Composite Resin

**FIGURE 3:**
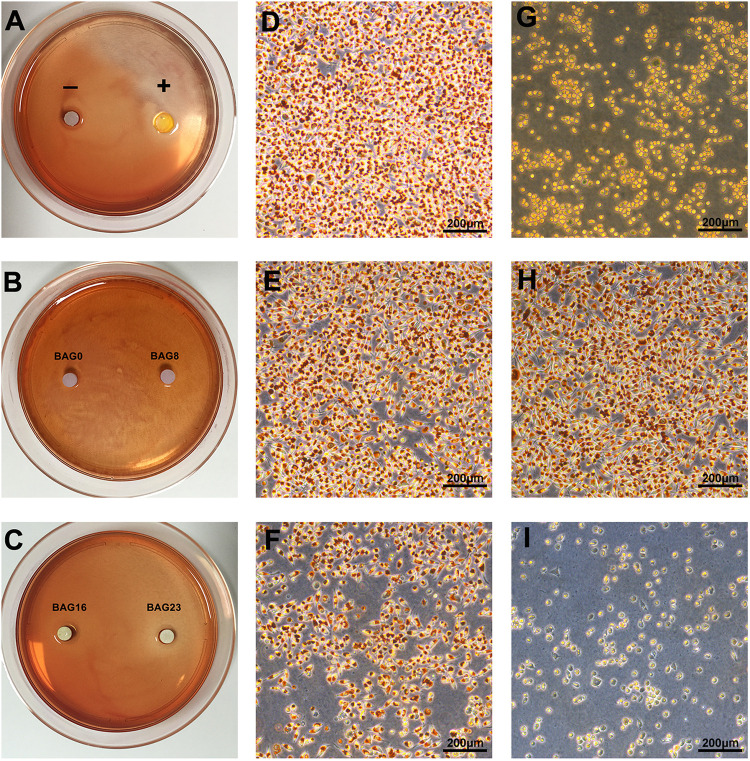
Cytotoxicity determined by agar diffusion test. **(A–C)** Representative gross images of decolorization zones in the agar diffusion test. **(D–I)** Representative microscope images of cellular decolorization and lysis in the agar diffusion test. Bar = 200 μm. **(D)** The negative control. **(E)** BAG0 group. **(F)** BAG16. **(G)** The positive control. **(H)** BAG8 group. **(I)** BAG23 group. “–” indicates the negative control (high-density polytetrafluoroethylene sheet); “+” indicates the positive control (polyvinylchloride containing organotin additives sheet); BAG0 indicates composite resin without BAG; BAG8 indicates composite resin with 8 wt% BAG; BAG16 indicates composite resin with 16 wt% BAG; BAG23 indicates composite resin with 23 wt% BAG.

According to ISO 10993.5 (Standardization IOF, 2009), an *in vitro* cytotoxicity assay is the first test to evaluate the biocompatibility of biomaterials for medical use. Therefore, two test methods, the agar diffusion test and MTT assay, were used in this study. In the agar diffusion test, the cytotoxic potential of the leachable contents of the test samples was determined after diffusion through an agarose layer by scoring the decolorization area and assessing cell lysis of the cell culture. The decolorization zones in the different groups are presented in Figure 3. The cytotoxicity scoring grade based on the decolorization index and the cell lysis index for the different groups are shown in Table 5. The negative control showed no cellular decolorization and lysis, while the positive control caused severe cellular decolorization and lysis with a cytotoxicity score of 3. In the various experimental composite resin groups, increasing the amount of BAG fillers from 0 to 23 wt % resulted in a progressive increase in the values of the decolorization index and the lysis index. The BAG8 group showed no cytotoxicity, while the BAG16 group had slight cytotoxicity with a cytotoxicity score of 1, and the BAG23 group had moderate cytotoxicity with a cytotoxicity score of 2 (Table 5).

**TABLE 5:** Results of the agar diffusion test.

**Test materials**	**The decolorization index**	**The cell lysis index**	**Cell response**	**Cytotoxicity scoring grade**	**Interpretation**
BAG0	0	0	0	0	Non-cytotoxic
BAG8	0	0	0	0	Non-cytotoxic
BAG16	1	0	1	1	Slightly cytotoxic
BAG23	3	3	3	2	Moderately cytotoxic
Negative control	0	0	0	0	Non-cytotoxic
Positive control	4	5	4	3	Severely cytotoxic

**FIGURE 4:**
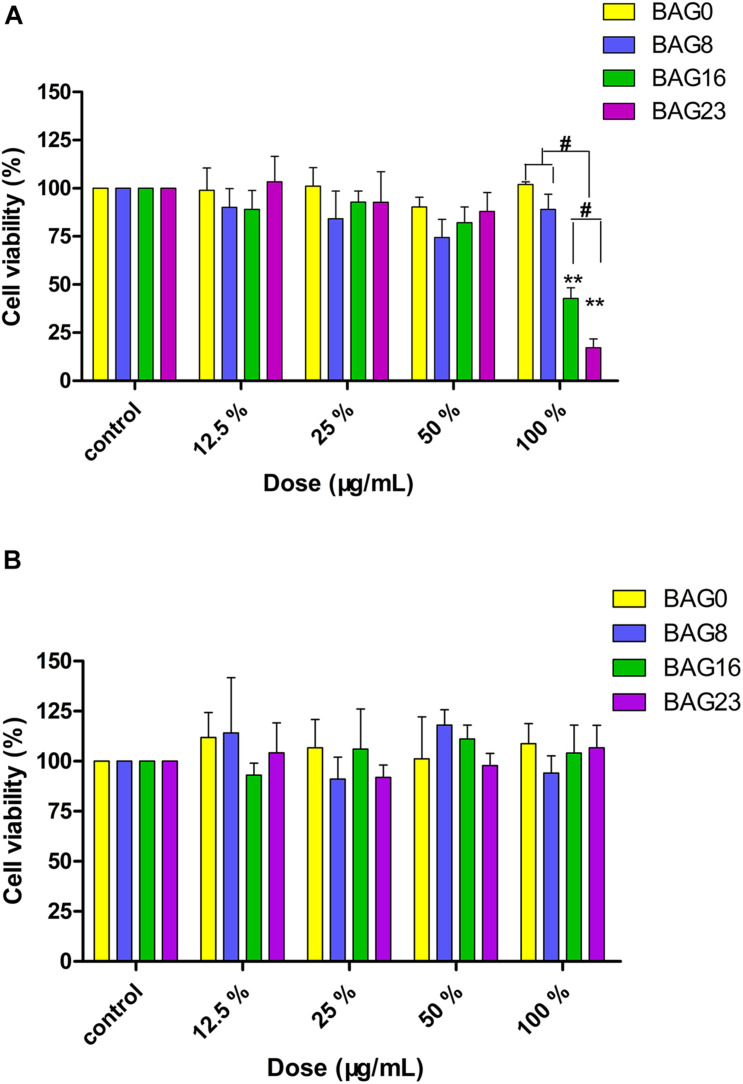
Cytotoxicity determined by MTT assay. **(A)** Cytotoxic effects of the extracts of BAG-modified composite resins after 24-h exposure. **(B)** Effects of pH adjustment of the extracts on cell viability. Results are presented in mean ± SEM of three independent experiments. **p < 0.01 vs. control. #*p* < 0.05 significant difference as compared groups. BAG0: the extracts of the composite resin without BAG; BAG8: the extracts of the composite resin with 8 wt% BAG; BAG16: the extracts of the composite resin with 16 wt% BAG; BAG23: the extracts of the composite resin with 23 wt% BAG.

Furthermore, the MTT assay was used to study the effect of the extracts from the BAG-modified composite resins on cell viability. As shown in Figure 4A, 12.5–50% extracts of all BAG-modified composite resins did not significantly alter cell viability when compared to the negative control, while 100% extracts of the composite resins with 16 or 23% BAG incorporation significantly reduced cell viability until the addition of a decrease to 8%. However, no concentration-dependent increase in cytotoxicity was observed in the presence of the extracts of all BAG groups. To clarify the role of alkaline pH values in the cytotoxicity of BAG-modified composite resins, the pH value of the extracts of all BAG groups was adjusted to neutral. Interestingly, following exposure to the neutral 100% extracts of BAG16 and BAG23, the cell viability became equal to the cell viability of the control (Figure 4B), suggesting that the cytotoxicity of BAG-modified composite resins might be attributed primarily to the increase in extracellular pH values.

### Antimicrobial Activity and Ion Release of BAG-Modified Composite Resin

**FIGURE 5:**
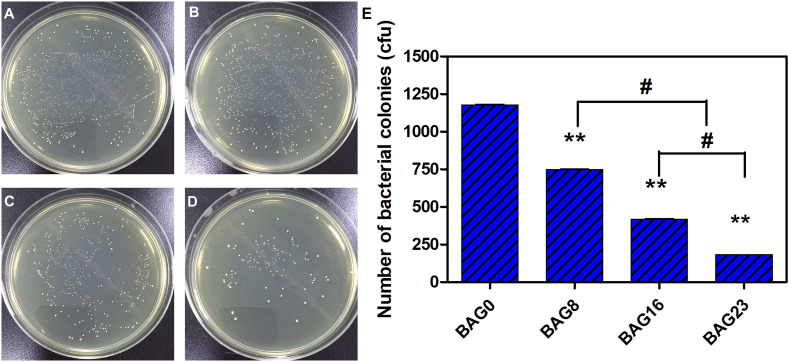
Direct antibacterial effect of modified composite resin with different mass fractions of BAG particles. **(A–D)** Representative gross images of *S. mutans* seeded on the BHI agar plate. **(A)** BAG0 group. **(B)** BAG8 group. **(C)** BAG16 group. **(D)** BAG23 group. **(E)** Bar graph shows quantification and statistical data of colony-forming units (CFUs) of bacterial colonies after different treatments. Data are shown as mean ± SEM, *n* = 3. BAG0 indicates composite resin without BAG; BAG8 indicates composite resin with 8 wt% BAG; BAG16 indicates composite resin with 16 wt% BAG; BAG23 indicates composite resin with 23 wt% BAG. **p < 0.01 vs. control. ^#^*p* < 0.05 significant difference as compared groups.

**FIGURE 6:**
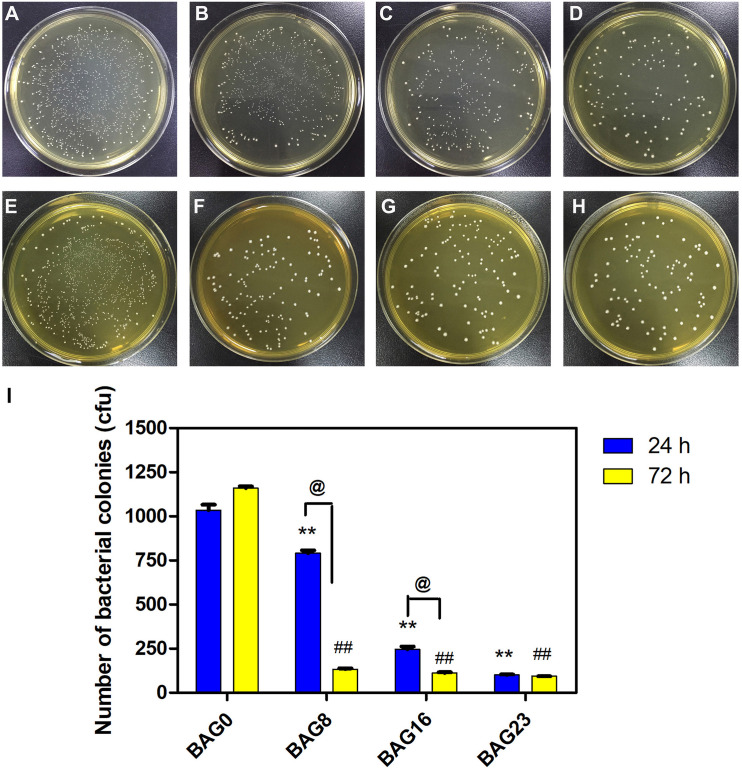
The antibacterial effect of the extracts of modified composite resin with different mass fractions of BAG particles. **(A–H)** Representative gross images of *S. mutans* seeded on the BHI agar plate after exposure to the 24-h extracts of **(A)** BAG0 group, **(B)** BAG8 group, **(C)** BAG16 group, and **(D)** BAG23 group, and the 72-h extracts of **(E)** BAG0 group, **(F)** BAG8 group, **(G)** BAG16 group, and **(H)** BAG23 group. **(I)** Bar graph shows quantification and statistical data of colony-forming units (CFUs) of bacterial colonies after different treatments. Data are shown as mean ± SEM, *n* = 3. BAG0 group as the control. *p < 0.05, **p < 0.01 vs. the 24 h extracts of BAG0 group, ^#^*p* < 0.05, ^##^*p* < 0.01 vs. the 72 h extracts of BAG0 group, ^@^*p* < 0.05 significant difference as compared groups. BAG0 indicates composite resin without BAG; BAG8 indicates composite resin with 8 wt% BAG; BAG16 indicates composite resin with 16 wt% BAG; BAG23 indicates composite resin with 23 wt% BAG.

The direct antibacterial effect of various BAG-modified composite resins was determined by counting bacterial colonies. As shown in Figure 5, after growing on the surface of BAG-modified composite resin for 24 h, the number of *S. mutans* colonies was significantly lower than the number of *S. mutans* colonies of unmodified composite resin (*p* < 0.01). In addition, a dose-dependent decrease in bacterial colonies was observed in the modified composite resins containing various amounts of BAG microparticles, suggesting that the addition of BAG can effectively improve the antibacterial performance of the composite resin. Moreover, to clarify whether the antibacterial effect depends on indirect action by soluble factors, various composite resin samples were extracted in bacterial culture medium for 24 and 72 h. Figure 6 indicates that 24 h of extracts from composite resins incorporated with different concentrations of BAG could obviously inhibit the growth of *S. mutans* in a concentration-dependent manner. The difference in the average number of bacterial colonies between composite resins with various amounts of BAG was statistically significant (*p* < 0.01). Similar inhibition was also observed in the groups treated with 72 h of extracts from BAG-modified composite resins, and the antibacterial effect in the 72-h extract group was significantly stronger than the antibacterial effect of the 24-h extract group (*p* < 0.05).

**FIGURE 7:**
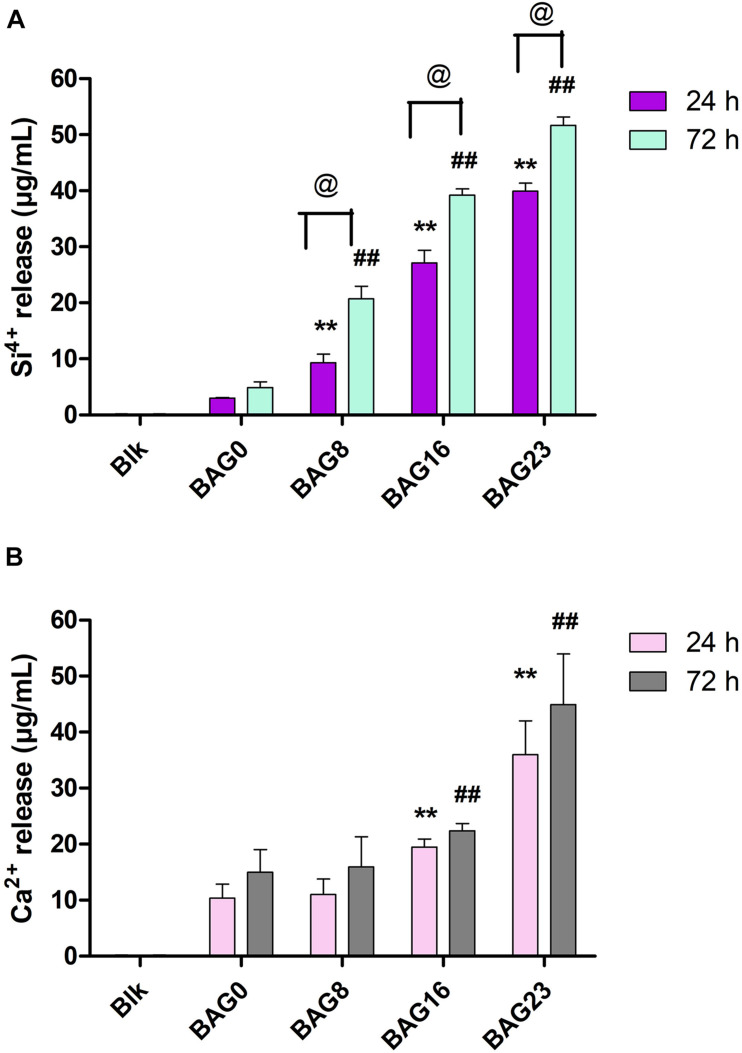
The concentration of Si^4+^ and Ca^2+^ in the BHI extracts of BAG-modified composite resin measured by inductively coupled plasma mass spectrometry (ICP-MS). **(A)** Si^4+^ release. **(B)** Ca^2+^ release. Blk: BHI as blank control; BAG0 indicates composite resin without BAG; BAG8 indicates composite resin with 8 wt% BAG; BAG16 indicates composite resin with 16 wt% BAG; BAG23 indicates composite resin with 23 wt% BAG. **p < 0.01 vs. the 24 h extracts of BAG0 group, ^##^*p* < 0.01 vs. the 72 h extracts of BAG0 group, ^@^*p* < 0.05 significant difference as compared groups.

The amounts of Ca and Si ions in the BHI extracts of each type of composite resin were determined and are presented in Figure 7. Si^4+^ release from the 24-h BHI extracts of experimental composite resins increased from 3.03 ± 0.12 μg/ml to 39.93 ± 1.45 μg/ml with the addition of BAG from 0 to 23 wt %, and Ca^2+^ release from the 24-h extracts increased from 10.37 ± 2.50 μg/ml to 35.97 ± 6.03 μg/ml with BAG incorporation from 0 to 23 wt %. In addition, there were significant differences in Si ion content between the 24-h extracts from BAG-modified composite resins and the 72-h extract group (*p* < 0.05), while no obvious difference was observed in Ca^2+^ release between the 24-h extract group and the 72-h extract group (*p* > 0.05).

**FIGURE 8:**
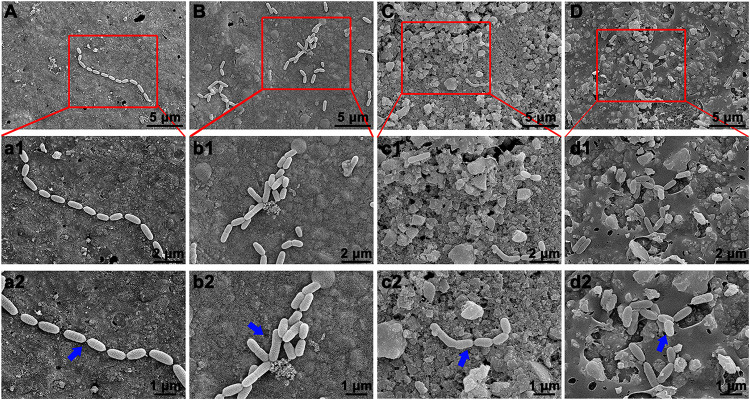
Bacterial morphology of *S. mutans* on surface of BAG-modified composite resin detected by scanning electron microscope (SEM). **(A,a1,a2)** BAG0 group; **(B,b1,b2)** BAG8 group; **(C,c1,c2)** BAG16 group; **(D,d1,d2)** BAG23 group. **(A–D)** Overall bacterial morphology (scale bar: 10 μm). **(a1–d1, a2–d2)** Higher magnification of the red box area in panels **(A–D)** (**a1–d1**, scale bar: 5 μm; **a2–d2**, scale bar: 3 μm). The blue arrow indicates *S. mutans*. BAG0 indicates composite resin without BAG; BAG8 indicates composite resin with 8 wt% BAG; BAG16 indicates composite resin with 16 wt% BAG; BAG23 indicates composite resin with 23 wt% BAG.

The bacterial adhesion and morphology of *S. mutans* on the surface of various composite resins was determined by SEM. Figure 8 reveals that *S. mutans* adhered well to the surface of unmodified composite resin, and the bacterial morphology was not changed, showing a typical and well-ordered chain-like arrangement. By contrast, the bacterial morphology and structure of *S. mutans* adhered to the surface of the BAG-modified composite resins obviously changed with increasing mass ratio of BAG incorporated into composite resins.

### Remineralizing Capability of BAG-Modified Composite Resin

**FIGURE 9:**
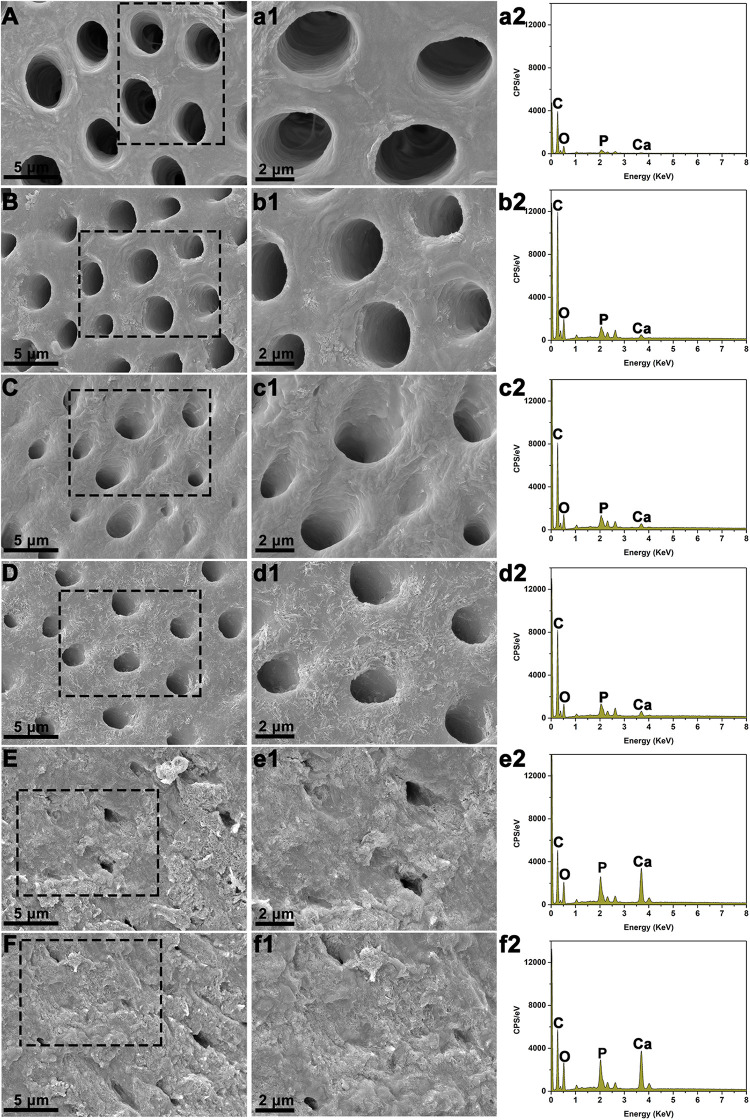
Scanning electron microscopy (SEM) with energy-dispersive X-ray spectroscopy (EDS) analysis of dentin surface morphology following immersion with various BAG-modified composite resins in SBF for 21 days. **(A,a1,a2)** The dentin disks with demineralized treatment immediately. **(B,b1,b2)** The demineralized dentin disks immersed in the blank SBF for 21 days. **(C,c1,c2)** The demineralized dentin disks immersed with BAG0 in the SBF for 21 days. **(D,d1,d2)** The demineralized dentin disks immersed with BAG8 in the SBF for 21 days. **(E,e1,e2)** The demineralized dentin disks immersed with BAG16 in the SBF for 21 days. **(F,f1,f2)** The demineralized dentin disks immersed with BAG23 in the SBF for 21 days. **(A-F)** SEM images (scale bar: 5 μm); **(a1–f1)** Higher magnification of the black box area in panels **(A–F)** (scale bar: 2 μm); **(a2–f2)** EDS analysis. BAG0 indicates composite resin without BAG; BAG8 indicates composite resin with 8 wt% BAG; BAG16 indicates composite resin with 16 wt% BAG; BAG23 indicates composite resin with 23 wt% BAG.

After immersing various BAG-modified composite resins with demineralized dentin disks in SBF for 21 days, the surface microstructure and elemental analysis of the dentin were observed by SEM-EDS. The uniform and smooth morphology of dentin was observed immediately after demineralized treatment or immersion in the blank SBF for 21 days (Figures 9A,B). Additionally, dentin tubules were clearly visible on the surface of demineralized dentin in the unmodified composite resin group (Figure 9C). By contrast, after immersion with various BAG-incorporated composite resins in SBF for 21 days, with increasing BAG amounts, an increasing number of dentin tubules were occluded by the mineralized layer (Figures 9D–F). According to the EDX analysis, a completely demineralized dentin surface immersed in SBF for 21 days showed that calcium was rarely observed. However, with the increase in the content of BAG incorporation, the amounts of calcium and phosphate gradually increased on the dentin surface, indicating that occluding deposits observed in each experimental group were calcium-rich mineralized layers and showing that BAG-modified composite resins have good remineralizing properties.

## Discussion

The present study successfully developed a novel bioactive hybrid composite resin with antibacterial and remineralizing activity by incorporating different weight fractions of BAG particles. SEM-EDS mapping indicated that BAG particles were homogeneously distributed in the resin matrix (Figure 1). Mechanical properties such as compressive strength, flexural strength, and microhardness are important for load-bearing applications of composite resin; thus, incorporation of BAG particles into the composite resin to achieve antibacterial and remineralizing functions should not sacrifice the mechanical properties of the composite resin. In this study, the flexural strength of the experimental composite resin containing 0 to 23 wt % BAG particles fulfilled the requirement of ISO 4049 for a minimum flexural strength of 80 MPa (Figure 2A; Standardization IOF, 2019). Moreover, our findings further showed that increasing the amount of BAG particles did not affect the short-term compressive and flexural strengths of the composite resin and resulted in only a slight decline in microhardness (Figures 2A–C), which is consistent with other previous studies reporting that the mechanical properties did not differ among various composite resins containing BAG particles with 0–15 wt % (Khvostenko et al., 2013). Moreover, a previous study also reported that dental resins containing biostable glass filler (SiO_2_, Ba_2_O_3_, and Al_2_O_3_) had higher hardness than the dental resins containing BAG filler due to their higher resistance to wear (Proença et al., 2020), suggesting that the decrease in microhardness of BAG-modified composite resins might be attributed to a lower ratio of barium glass filler. By contrast, a continuous reduction in the flexural strength and modulus of the composite resin was observed with increasing BAG content and aging (Yang et al., 2013; Par et al., 2019b). Generally, the mechanical properties of composite resins were largely dependent on polymerization of the resinous matrix, inorganic fillers and filler/matrix interface (Par et al., 2019b). BAG fillers have been reported to have a direct inhibitory effect on the polymerization of Bis-GMA resin systems because of surface oxides (Par et al., 2019a,b), but our data showed that the DC for the as-prepared resin system was not compromised by the addition of BAG fillers up to 23 wt % (Figure 2F). A possible explanation is that the larger BAG microparticles used in our study had fewer surface oxides than the smaller BAG particles in other studies (Par et al., 2019b). Moreover, incorporating a low content of larger BAG microparticles (∼7.26 μm) and a high content of smaller barium glass microparticles (∼0.7 μm) might increase the fracture toughness of the composite resin by crack deflection or bridging (Khvostenko et al., 2013; Kundie et al., 2018). Thus, the mechanism underlying the negligible effect of BAG incorporation on compressive and flexural strengths might be attributed to a higher content of hybrid inorganic fillers and a higher DC of resin matrix.

Investigation of the biocompatibility of the novel composite resin is a pre-requisite before clinical use. In compliance with the ISO standards (Standardization IOF, 2009, 2011), both the agar diffusion test and MTT assay were selected to evaluate the cytotoxicity of dental composite resins based on different endpoints in this study. Specifically, the agar diffusion test, as a well-established cytotoxicity barrier test in dentistry, is commonly used to determine cell membrane integrity by mimicking the penetration of leachable substances released from dental materials through the dentin/mucosal membrane barrier. The MTT assay was used to detect mitochondrial activity by simulating the leachable constituents extracted from dental materials (Kilic et al., 2012). The agar diffusion test results showed that the cytotoxicity score of the experimental composite resins with a low (8–16 wt %) mass fraction of BAG filler was grade 0–1, which falls within the acceptable range for medical devices (Standardization IOF, 2009, 2011), but when the mass ratio of BAG filler increased up to 23%, the cytotoxicity score was grade 2, indicating increasing cell membrane permeability with increasing BAG incorporation (Table 5). Additionally, the cell metabolic activity could be reduced significantly by exposure to the extracts of composite resins with 16–23 wt % BAG fillers (Figure 4A). In other previous studies, the major reason for cytotoxicity was attributed predominantly to the release of residual monomers, not to the presence of the BAG fillers (Salehi et al., 2015; Balhaddad et al., 2019). However, our data showed that neither the unmodified composite resins nor the modified composite resins with 8 wt % BAG exhibited cytotoxicity, reflecting that the level of residual monomers released from our experimental resin matrix would not be sufficient to cause cytotoxic effects. As the degradation of BAG was accompanied by the release of alkali metal ions and an increase in pH in the local microenvironment, a higher pH value might be the prominent factor affecting the cytotoxicity of BAG-modified composite resins. The range of the optimal pH values for cell growth is considered to be between 7.4 and 7.8 (Wang et al., 2015). However, the pH values in the extracts of the four experimental composite resins reached 9.0 when the addition of BAG was 16–23 wt % (Figure 2D). Because the *in vitro* cell culture system lacks the buffering capacity of the *in vivo* environment, the pH value of the *in vitro* test system could be adjusted to the normal level to simulate the *in vivo* environment (Wang et al., 2015; Willbold et al., 2017). After adjusting the pH value of the extracts of BAG16 and BAG23 to 7.4, the cell viability recovered significantly up to 100% above (Figure 4B).

As composite resins were usually shown to accumulate more biofilms and plaque than other restorative materials (Singh et al., 2009), it is highly desirable to develop composite resins with antibacterial activity to inhibit bacteria and combat secondary caries. Many previous studies, including ours, have found that BAG particles have strong antibacterial effects on a variety of bacteria and biofilms due to ion release (e.g., Ca, P, Na, and Si ions) and the pH elevation of the surrounding environment (Xu et al., 2015; Kargozar et al., 2018; Bauer et al., 2019). BAG particles were also reported to be able to insert into bacterial membranes like a needle, leading to the destruction of the cell walls of bacteria (Hu et al., 2009). However, it is still not clear whether this antibacterial behavior could be affected when the BAG particles were embedded in the composite resin matrix as a filler. In the present study, *S. mutans*, as one of the major caries-related species, was chosen to represent the dominant streptococci genus in dental biofilms. Our data showed that all the composites containing BAG particles significantly reduced the CFU of *S. mutans* and inhibited bacterial growth on the surface of composite resins (Figure 5), consistent with other studies, which showed that the addition of BAG particles into composite resins yielded a significant inhibition of *Escherichia coli* and *S. mutans* and reduced the bacterial penetration depth into the interface tooth resins (Khvostenko et al., 2013, 2016; Chatzistavrou et al., 2015). To explore the role of direct surface contact or soluble factor release in its antibacterial action, each composite resin sample with the same specification in the above direct contact antibacterial experiment was extracted in bacterial culture medium for 24 and 72 h, and then the extracts were used to culture *S. mutans* for 24 h. The results showed that the 72-h extracts of various BAG-modified composite resins had better antibacterial effects than the 24-h extract groups with obvious dose- and time-dependent bacterial inhibition (Figure 6). Correspondingly, we found that the antibacterial effects of the extracts of BAG-modified composite resins were higher than the antibacterial effects of the direct contact groups at the same aqueous pH value (Figures 5, 6), suggesting that the antibacterial mechanism of the BAG-modified composite resin was attributed dominantly to the cumulative effects of ion release, not just the increase in pH value or direct surface contact. This conclusion was further supported by the results from the pH measurements and the ion release in the BHI extracts. Figure 2E shows that the pH values in the extracts of BAG-modified composite resin groups were approximately 8.04–8.87, which fell within the *S. mutans*-tolerated pH range of 4–10 described in the literature (Nakajo et al., 2006). Based on ICP-MS analysis, a significant dose-dependent increase in the release of Si^4+^ and Ca^2+^ was observed in the BHI extracts of experimental composite resins with increasing BAG amounts, especially the Si ions released from the composite resin with 23 wt % BAG, which were more than 10 times higher than the Si ions released from the control without BAG (Figure 7), suggesting that the combined action of Si^4+^ and Ca^2+^ release is the main factor contributing to the antibacterial properties of BAG-modified composite resin. The release of Si^4+^ or Ca^2+^ from BAG has also been suggested to prohibit bacterial growth by causing perturbations of the membrane potential of bacteria or attacking target sites on the cell membranes of bacteria in many previous studies (Keenan et al., 2017; Drago et al., 2018; Aponso et al., 2019; Dai et al., 2020). Consistently, our SEM data further showed that on the surface of composite resins containing 8 and 16 wt % BAG, the long-chain-like structure of *S. mutans* gradually disintegrated into a short-chain shape. Especially on the surface of composite resins containing 23 wt % BAG, the long-chain structure basically disappeared, indicating that the incorporation of BAG could not only reduce the number of bacteria adhered to the composite resin surface but also disturb the morphology and structure of bacteria (Figure 8).

Secondary caries could lead to dentin demineralization by bacterial acids in carious lesions; therefore, in addition to the antibacterial ability, the ideal composite resin should have excellent mineralizing capability to inhibit secondary caries and thus extend the lifetime of restoration. BAG has been proven to promote dentin remineralization in many previous studies (Bakry et al., 2014a,b; Mehta et al., 2014), so BAG has been added to toothpaste, anti-carious gel and dentin desensitizer for clinical use. Once BAG particles were used to modify the composite resin, it was critical that these particles embedded in the resin matrix could continue to exert bioactivity. A previous study reported that the hydroxylapatite layer could be formed on the surface of dental adhesive containing 20 wt % BAG particles after immersion in PBS for 21 days (Taubock et al., 2014), but the direct effects of BAG-modified composite resins on dentin remineralization were rarely highlighted. In this study, the capability of inducing remineralization was evaluated by exploring the surface structure and chemical composition of demineralized dentin surfaces of tooth slices mixed with BAG-modified composite resins upon soaking in SBF for 21 days. Our SEM results (Figure 9) showed that the demineralized dentin surfaces were covered with a Ca-P-containing layer following immersion with 16–23 wt % BAG-modified composite resins in SBF solutions, while the dentin surfaces showed clearly visible dentin tubules in the control without BAG-modified composite resins. In addition, with increasing amounts of BAG particles within the composite resin, the amounts of Ca and P gradually increased on the demineralized dentin surface, indicating that the incorporation of the BAG particles into the resin matrix could also exert bioactivity and promote dentin remineralization. Materials containing released Ca, P, and Si ions have been widely accepted to be favorable for remineralization (Cheng et al., 2017; Fernando et al., 2017). A previous study focused on the effects of the addition of BAG in polymer constructs on bioactivity and found that the precipitation of mineralized layers on the surface of materials was ascribed to a well-defined ion exchange mechanism between metal cations (Na^+^ and Ca^2+^) in the BAG and hydronium ions (H_3_O^+^) in the surrounding fluid (Serio et al., 2019). Therefore, in SBF solution, the enhanced remineralization on the demineralized dentin surface was attributed mainly to the release of bioactive ions from BAG and the change in the local microenvironment. Taken together, the above data suggest that the BAG-modified composite resin has both remineralizing and antibacterial capabilities, which could be regulated by the controllable release of bioactive ions (Si^4+^ and Ca^2+^).

There are several limitations of this study. First, the decline in mechanical properties with aging has been reported in a previous study (Par et al., 2019b); thus, testing the mechanical properties of composite resins immersed in water for a long time (e.g., 30, 60, or 90 days) might be more suitable for the evaluation of the mechanical properties of the material. Second, it would be more appropriate to detect the antibacterial effects of BAG-modified composite resin on biofilms and investigate their remineralization ability using *in situ* or *in vitro* models under clinically relevant conditions, which would make the conclusions on the antibacterial and remineralizing properties more accurate.

## Conclusion

In summary, a novel BAG-modified hybrid composite resin was developed with excellent biocompatibility and antibacterial and remineralizing capabilities. When the mass fractions of BAG particles were added from 8 to 23 wt %, the original mechanical properties of the composite resin, including flexural strength and compressive strength, were not obviously affected without compromising the DC. Although the BAG incorporation of mass fractions of 16–23 wt % in composite resins reduced cell viability, the cell viability could be recovered by the regulation of pH values. The synthesized BAG-modified composite resins showed good antibacterial effects against *S. mutans* and enhanced remineralization activity on demineralized dentin surfaces in a concentration-dependent manner. The possible mechanisms of antibacterial activity and dentin remineralization might be closely related to the release of bioactive ions, suggesting that the BAG antibacterial and biological properties can be controlled by regulating the amounts of bioactive ions. The capability to balance the mechanical properties, cytotoxicity, antibacterial activity, and bioactivity makes BAG-modified composite resin a promising prospect for clinical application.

## Data Availability Statement

The original contributions presented in the study are included in the article/supplementary material, further inquiries can be directed to the corresponding author/s.

## Ethics Statement

The studies involving human participants were reviewed and approved by Ninth People’s Hospital, Shanghai Jiao Tong University School of Medicine. The patients/participants provided their written informed consent to participate in this study.

## Author Contributions

XH: conceptualization, methodology, investigation, and writing – original draft. YC: methodology, investigation, and formal analysis. QJ: methodology, writing – review and editing, and funding acquisition. XL: conceptualization, project administration, and funding acquisition. YmC: supervision, resources, project administration, and funding acquisition. All authors contributed to the article and approved the submitted version.

## Conflict of Interest

The authors declare that the research was conducted in the absence of any commercial or financial relationships that could be construed as a potential conflict of interest.
